# Postpartum maternal depression, mother-to-infant bonding, and their association with child difficulties in sixth grade

**DOI:** 10.1007/s00737-025-01585-y

**Published:** 2025-04-15

**Authors:** Daimei Sasayama, Tomonori Owa, Tetsuya Kudo, Wakako Kaneko, Mizuho Makita, Rie Kuge, Ken Shiraishi, Tetsuo Nomiyama, Shinsuke Washizuka, Hideo Honda

**Affiliations:** 1 Department of Psychiatry, Shinshu University School of Medicine, 3 - 1- 1, Asahi, Matsumoto, Nagano, 390 - 8621 Japan; 2 Department of Child and Adolescent Developmental Psychiatry, Shinshu University School of Medicine, Matsumoto, Nagano, 390 - 8621 Japan; 3 Mental Health Clinic for Children, Shinshu University Hospital, Matsumoto, Nagano, 390 - 8621 Japan; 4 Shinano Medical Welfare Center, Shimo-Suwa, Nagano, 393 - 0093 Japan; 5 Department of Preventive Medicine and Public Health, Shinshu University School of Medicine, Matsumoto, Nagano, 390 - 8621 Japan

**Keywords:** Child Behavior, Longitudinal Study, Mother–Child Interactions, Postpartum Depression

## Abstract

**Purpose:**

Postpartum maternal mental health plays a crucial role in the development of children’s social and emotional competencies. This study aimed to investigate the influence of postpartum maternal depression and mother-to-infant bonding on children’s emotional and behavioral difficulties in sixth grade.

**Methods:**

Data from the maternal Edinburgh Postnatal Depression Scale (EPDS) and the Mother-to-Infant Bonding Scale-Japanese version (MIBS-J), administered to mothers approximately 2 weeks to 1 month postpartum during postnatal health checkups in Okaya, Japan, were analyzed alongside Strengths and Difficulties Questionnaire (SDQ) data collected from their sixth-grade children and their caregivers. The study included 245 mother–child pairs of children born between April 2, 2009, and April 1, 2012.

**Results:**

Postpartum maternal depressive symptoms, as assessed by the EPDS, were significantly associated with mother-to-infant bonding difficulties, as assessed by the MIBS-J. Structural equation modeling revealed that EPDS, MIBS-J, and sex significantly predicted psychosocial difficulties of children. Bonding difficulties mediated 34.6% of the total effect of EPDS on child difficulties. The models explained 26.1% of the variance in psychosocial difficulties, with 43.0% of the variance explained for parent-rated SDQ scores and 36.4% for self-rated SDQ scores.

**Conclusions:**

The negative impact of maternal depressive symptoms on mother-to-infant bonding may have contributed to increased difficulties for the child, highlighting the critical role of bonding in moderating the effects of maternal mental health on child development. These findings underscore the importance of early postpartum interventions targeting both maternal depression and bonding difficulties to mitigate long-term effects on child development.

**Supplementary Information:**

The online version contains supplementary material available at 10.1007/s00737-025-01585-y.

## Article Highlights

• Postpartum depression and mother-to-infant bonding predict child psychosocial difficulties in sixth grade.

• Postpartum depression in mothers is linked to bonding difficulties with their children.

• Bonding mediates 34.6% of the effect of postpartum maternal depression on child difficulties in sixth grade.

• The findings highlight the enduring associations of early bonding experiences with emotional and behavioral development.

**Supplementary Information:**

The online version contains supplementary material available at 10.1007/s00737-025-01585-y.

## Introduction

Child development and well-being are shaped by numerous factors beyond immediate parenting practices. Maternal mental health plays a significant role in offspring development and is a critical target for early intervention (Rogers et al. 2020). Infants of mothers with depression often exhibit difficult temperament (Netsi et al. 2015), disturbed sleep (O'Connor et al. 2007), and delayed cognitive development (Azak 2012; Cogill et al. 1986; Conroy et al. 2012). These behavioral challenges can, in turn, exacerbate maternal depression (Dix and Yan 2014).

Mother-to-infant bonding is a multifaceted construct that refers to the deep emotional attachment between a mother and her infant. It is considered essential for a child’s optimal social and emotional development (Crouch and Manderson 1995). The impact of postpartum mental health on mother-to-infant bonding is well-documented (Edhborg et al. 2011; Lutkiewicz et al. 2020; Taylor et al. 2005; Tietz et al. 2014). Specifically, postpartum depression can impede offspring development by reducing the mother’s sensitivity to infant cues, thereby diminishing the likelihood of secure infant attachment (Bakermans-Kranenburg et al. 2003). For example, postpartum depression adversely affects a mother’s ability to care for her infant and causes difficulties in responding to the baby through social interactions (Murray et al. 2003). Postpartum depression is also linked to premature cessation of breastfeeding, complicating maternal-infant interactions (Dennis and McQueen 2007).

While short-term effects of postpartum depression on early child development are well-documented (as highlighted in the systematic review by Slomian et al (2019)), less is known about its long-term impact on behavioral outcomes beyond middle childhood. For example, a weak association between maternal postpartum depression and children’s emotional problems at ages 11–12 was reported (Walker et al. 2020), while other studies found significant associations with internalizing problems at age six but not at age eight (Tainaka et al. 2022). Our prior research similarly identified a weak but significant association between maternal postpartum depressive symptoms and children’s attention and hyperactivity difficulties in sixth-graders (Sasayama et al. 2024). Moreover, a longitudinal study found that increase in maternal depression scores during pregnancy raised the offspring’s risk of depression at age 18 (Pearson et al. 2013). Although evidence above suggests that maternal postpartum depression is a significant risk factor for later mental health difficulties in children, the reported magnitude of this risk varies across studies. This inconsistency is likely due to the influence of confounding factors that are difficult to control, such as cultural differences, as well as the potential impact of unknown mediating factors that shape the relationship between maternal postpartum depression and child mental health difficulties.

Despite limited evidence on the long-term outcomes, secure mother-to-infant bonding has lasting positive effects on children’s social-emotional competencies (Behrendt et al. 2019; Joas and Mohler 2021; Rusanen et al. 2024). Attachment Theory underscores the importance of early bonding experiences in shaping a child’s emotional and social development. Parent–child interactions are inherently dynamic and bidirectional, with both parties influencing each other over time (Girard et al. 2017), as described in the Transactional Model of Development (Sameroff 2009). Maternal depression can disrupt these interactions, weakening the mother-to-infant bond and increasing the likelihood of insecure attachment. Insecure attachment styles, often associated with maternal depression, have been linked to developmental challenges such as anxiety (Muris and Meesters 2002) and depression (Abela et al. 2005) in children. Consequently, maternal depression not only contributes to insecure attachment but also adversely affects a child's emotional and cognitive development.

Evidence is scarce regarding how maternal depression and bonding difficulties jointly influence psychosocial outcomes in middle childhood. This study addresses this gap by proposing a theoretical framework that highlights the mediating role of mother-to-infant bonding and provides methodological insights for exploring long-term child development outcomes.

Research using the Strengths and Difficulties Questionnaire (SDQ) to assess behavioral difficulties in children in Japan has shown that boys tend to have higher difficulty scores than girls (Moriwaki and Kamio 2014). Additionally, differences between parent-rated and self-rated SDQ scores have been reported (Van Roy et al. 2010). Based on this evidence, we collected both parent-rated and self-rated SDQ data from sixth-grade children, and included sex as a predictor of SDQ in the statistical model.

Attachment Theory suggests that early maternal sensitivity is crucial for a child's emotional development, and disruptions in bonding due to postpartum depression may contribute to later mental health difficulties. The Transactional Model of Development further emphasizes bidirectional influences between maternal and child mental health (Sameroff 2009). Based on these frameworks, we hypothesize that mother-to-infant bonding mediates the relationship between maternal depression and child mental health outcomes over time. If mother-to-infant bonding serves as a mediator, accounting for its influence in statistical analyses could provide a clearer understanding of the direct and indirect effects of maternal depression on child difficulties while reducing the impact of confounding variables. Therefore, the present study aims to examine the relationship between maternal postpartum depression and child mental health outcomes, incorporating mother-to-infant bonding as a mediating factor to refine the analysis and enhance the accuracy of the findings.

## Materials and methods

A detailed description of methods is provided in the Supplementary Information. Okaya, a city in central Japan, was selected as the research setting. Edinburgh Postnatal Depression Scale (EPDS) and Mother-to-Infant Bonding Scale-Japanese version (MIBS-J) data from postnatal health checkups, approximately 2 weeks to 1 month after delivery, were analyzed for mothers of children born between April 2, 2009, and April 1, 2012. The Japanese version of parent- and self-rated SDQ were distributed to all sixth graders and their caregivers in public elementary schools in Okaya in November of 2021, 2022, and 2023. An information sheet detailing the study was enclosed with the questionnaire. Only caregivers who consented for both themselves and their child to participate were asked to complete the questionnaires and allow their children to do the same. The inclusion criteria were: (1) both child- and parent-completed SDQ data were available, and (2) the mother’s EPDS and MIBS-J data were accessible from the postpartum health checkup records. The exclusion criteria were: (1) participants with invalid SDQ data, and (2) children from multiple births. The study was approved by the ethics committee of the Shinshu University School of Medicine (approval number 5129).

Maternal depressive symptoms and mother-to-infant bonding were evaluated using the EPDS and MIBS-J, respectively, conducted during postnatal health checkups. Psychosocial difficulties in the children were assessed using the SDQ (Goodman 2001), completed by sixth-grade children and their caregivers.

MIBS-J and SDQ scores were treated as continuous variables. Path analysis using ‘lavaan’ (Rosseel 2012) was conducted to examine the relationships among sex, maternal postpartum depressive symptoms, mother-to-infant bonding, and difficulties in the sixth grade. The presence of maternal postpartum depressive symptoms and sex were entered as the predictor, MIBS-J scores as the mediator, and SDQ scores as the dependent variable. Additionally, we modeled self-rated and parent-rated SDQ scores as indicators of a single latent variable “psychosocial difficulties” to capture their shared variance, enabling a unified representation of child difficulties in the analysis. An additional analysis was performed with maternal age included as a covariate. All tests were two-tailed, and statistical significance was set at p < 0.05.

## Results

**Fig. 1:**
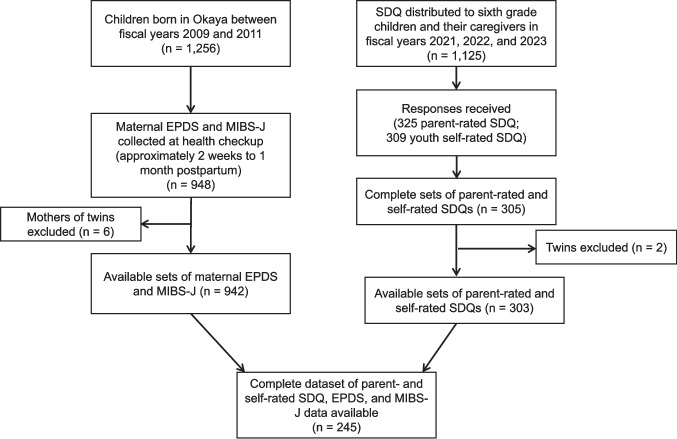
Flowchart of the inclusion procedure. The EPDS and MIBS-J data from postnatal checkups in Okaya were retrieved for 948 mothers who completed both questionnaires. The SDQ was distributed to 1,125 sixth grade children and their caregivers, with responses received from 325 caregivers and 309 children. A complete set of parent- and self-rated SDQ, EPDS, and MIBS-J data was available for 245 caregiver-child pairs. Abbreviations: EPDS, Edinburgh Postnatal Depression Scale; MIBS-J, Mother-to-Infant Bonding Scale-Japanese version; SDQ, Strengths and Difficulties Questionnaire

The EPDS and MIBS-J data from postnatal checkups in Okaya were retrieved for mothers of 954 children born between April 2, 2009, and April 1, 2012, who completed both questionnaires. After excluding mothers of twins, 942 sets of maternal EPDS and MIBS-J were available for analysis. Among these mothers, 145 (15.4%) had scores ≥ 9, meeting the cutoff score for depression. The mean (standard deviation [SD]) score for the MIBS-J was 1.8 (2.3), with 57.1% scoring 0 or 1 and 28.1% scoring 3 or above. The parent-rated and self-rated SDQs were retrieved from 245 of these 942 children and their caregivers (Fig. 1). Among the caregivers who completed the parent-rated SDQs, 226 (92.2%) were mothers, 18 (7.3%) were fathers, and one (0.4%) was another caregiver.

Table 1 shows the demographic and clinical characteristics of the 245 participants. Forty-two (17.1%) mothers from the analyzed 245 mother–child pairs scored 9 or above on the EPDS and were classified as having postpartum depressive symptoms. The mean (SD) score of the MIBS-J was 1.8 (2.4), with 58.0% scoring 0 or 1 and 26.5% scoring 3 or above. MIBS-J scores were significantly higher for mothers who had depressive symptoms than for those who did not (U = 2340.5, *p* < 0.001; mean [SD] = 3.6 [3.3], median = 3 for mothers with depressive symptoms and mean [SD] = 1.4 [1.9], median = 1 for mothers without).

**Table 1:** Demographic and clinical characteristics of participants

Maternal age at delivery^a^	32.3 (4.5) years
Maternal postpartum EPDS score above cutoff	17.1%
Maternal postpartum MIBS-J score	1.8 (2.4)
Sex	113 boys/132 girls
Firstborn child	41.6%
Birth weight	2987.1 (433.3) g
Gestational age at birth^b^	275.7 (10.3) days
Paternal cohabitation at birth	99.6%

Numbers are shown as mean (standard deviation) unless otherwise specified

*EPDS* Edinburgh Postnatal Depression Scale; *MIBS-J* Mother-to-Infant Bonding Scale-Japanese version;

^a^Maternal age at delivery data were available for 244 out of 245 participants (1 missing)

^b^Gestational age at birth data were available for 243 out of 245 participants (2 missing)

Supplementary Table 1 shows the attrition analysis comparing mothers included in the final analysis (*n* = 245, 26.0%) and those not included (*n* = 697, 74.0%). No significant differences were observed in MIBS-J scores or the proportions of mothers with EPDS scores above the cutoff.

Table 2 presents the correlation matrix of the key variables. Both parent-rated and self-rated SDQ scores showed significant correlation with EPDS and MIBS-J scores, as well as with the child’s sex. Additionally, lower gestational age at birth was significantly associated with EPDS above the cutoff and with higher MIBS-J scores but not with SDQ scores.

**Table 2:** Spearman's correlation matrix of key variables

	EPDS cutoff	MIBS-J	SDQ (parent-rated)	SDQ (self-rated)	Sex	Maternal age at delivery	Birth order	Birth weight	Gestational age
EPDS cutoff (0: below cutoff, 1: above cutoff)									
MIBS-J score	.305**								
SDQ (parent-rated)	.218**	.189**							
SDQ (self-rated)	.158*	.172**	.619**						
Sex (1: males, 2: females)	− 0.057	− 0.079	-.166**	-.220**					
Maternal age at delivery	0.094	0.100	0.079	.162*	− 0.081				
Birth order (1: Firstborns, 2: Laterborns)	− 0.018	− 0.012	− 0.091	− 0.069	.151*	− 0.005			
Birth weight	− 0.067	0.014	0.088	0.057	-.206**	0.091	.377**		
Gestational age at birth	-.196**	-.193**	− 0.026	0.038	− 0.077	.179**	− 0.072	.132*	

*EPDS* Edinburgh Postnatal Depression Scale, *MIBS-J* Mother-to-Infant Bonding Scale-Japanese version, *SDQ* Strengths and Difficulties Questionnaire

*p < 0.05, ** *p* < 0.01

**Fig. 2:**
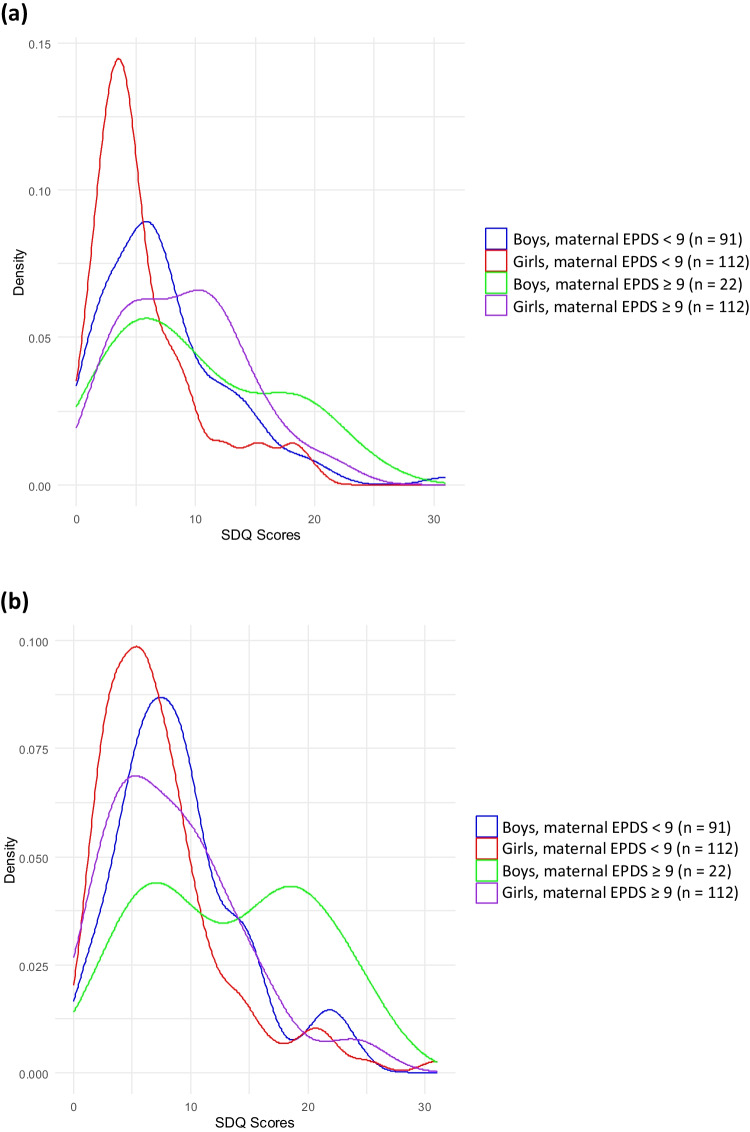
Distribution curves of SDQ scores in sixth grade children. The distribution curves of parent-rated SDQ scores (Fig. 2a) and self -rated SDQ scores (Fig. 2b) in sixth-grade children are shown. Both parent- and self-rated SDQ scores were significantly higher in children whose mothers had EPDS scores above the cutoff (both *p* < 0.001). Abbreviations: EPDS, Edinburgh Postnatal Depression Scale; SDQ, Strengths and Difficulties Questionnaire

Table 3 shows the SDQ scores for boys and girls. Boys had significantly higher total difficulty scores compared to girls for both parent- and self-rated SDQ (both *p* < 0.001). Self-rated SDQ scores were significantly higher than parent-rated SDQ scores (z = − 5.35, *p* < 0.001). Figure 2a and b show the distribution curves of the SDQ scores. Both parent- and self-rated SDQ scores were higher in children whose mothers had maternal depressive symptoms (both *p* < 0.001). MIBS-J scores were significantly positively correlated with both parent- and self-rated SDQ scores (*ρ* = 0.189, *p* = 0.003 and *ρ* = 0.172, p = 0.007, respectively). No significant difference between boys and girls was observed in their mothers’ MIBS-J scores (U = 6796.0, *p* = 0.215) or in the proportion of mothers with EPDS scores ≥ 9 (*χ*^2^ = 0.799, *p* = 0.371).

**Table 3:** Strengths and Difficulties Questionnaire scores

	Boys (*n* = 113)	Girls (*n* = 132)	*P* value
Parent-rated			
Total Difficulties	7.9 (5.7)	6.2 (4.7)	0.010
Emotional symptoms	1.8 (2.1)	1.6 (2.3)	n.s
Conduct problems	1.7 (1.5)	1.2 (1.2)	0.007
Hyperactivity/inattention	2.8 (2.3)	1.8 (1.7)	0.001
Peer problems	1.7 (1.8)	1.7 (1.5)	n.s
Prosocial behavior	6.7 (2.1)	7.1 (1.9)	n.s
Self-rated			
Total Difficulties	9.9 (5.9)	7.6 (5.4)	< 0.001
Emotional symptoms	2.8 (2.4)	2.5 (2.5)	n.s
Conduct problems	1.8 (1.4)	1.3 (1.4)	0.003
Hyperactivity/inattention	3.2 (2.3)	2.1 (1.8)	< 0.001
Peer problems	2.1 (1.7)	1.7 (1.6)	0.013
Prosocial behavior	6.4 (2.0)	6.7 (2.1)	n.s

Numbers are shown as mean (standard deviation)

**Fig. 3:**
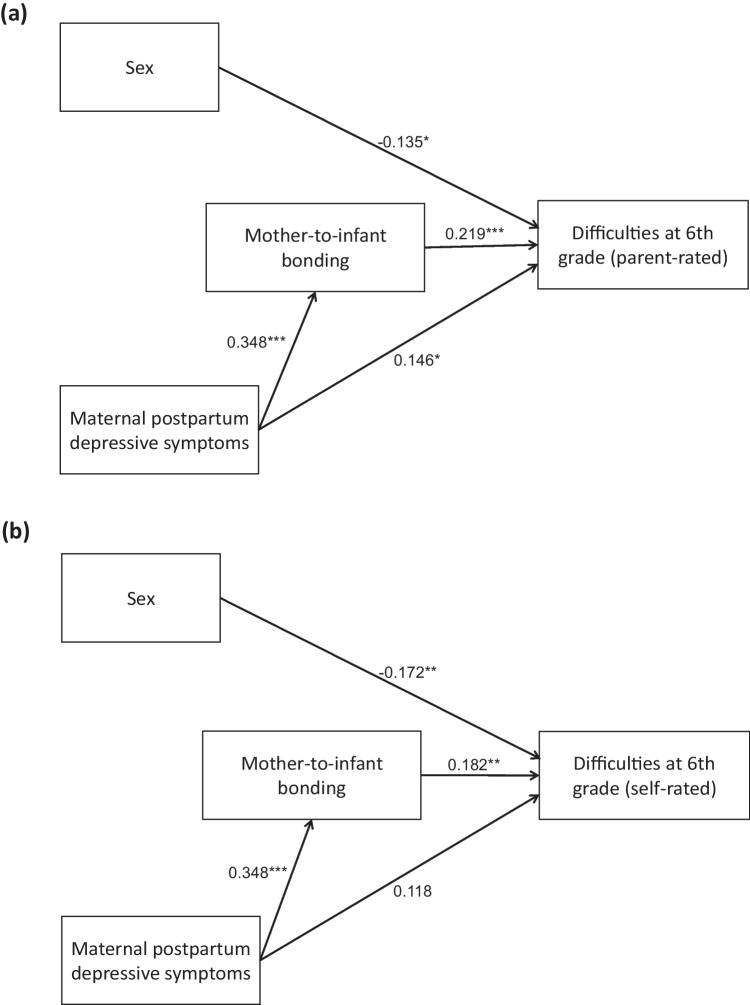
Path analysis. Figure 3a: Path analysis showing the effects of sex, maternal postpartum depressive symptoms, and mother-to-infant bonding on difficulties at sixth grade, as measured by the parent-rated Strengths and Difficulties Questionnaire (SDQ) (*n* = 245). The structural equation model demonstrated an acceptable fit to the data. The chi-square statistic was non-significant (*χ*^2^ = 1.519, df = 1, *p* = 0.218), indicating no substantial discrepancy between the model and the observed data. The comparative fit index (CFI = 0.991) and Tucker-Lewis index (TLI = 0.956) suggested a good fit, while the root mean square error of approximation (RMSEA = 0.046) and standardized root mean square residual (SRMR = 0.024) indicated a close approximation of the model to the data. The model explained 11.3% of the variance in SDQ scores and 12.1% of the variance in mother-to-infant bonding scores, as reflected in the *R*^2^ values for these outcomes. Figure 3b: Path analysis showing the effects of sex, maternal postpartum depressive symptoms, and mother-to-infant bonding on difficulties at sixth grade, as measured by the self-rated SDQ (*n* = 245). The structural equation model demonstrated an acceptable fit to the data, with *χ*.^2^ = 1.519 (df = 1, *p* = 0.218), CFI = 0.990, TLI = 0.952, RMSEA = 0.046, SRMR = 0.024, and the model explained 9.5% of the variance in SDQ scores and 12.1% of the variance in MIBS-J scores. Values shown in the figure are standardized estimates. *p < 0.05, **p < 0.01, ***p < 0.001

**Fig. 4:**
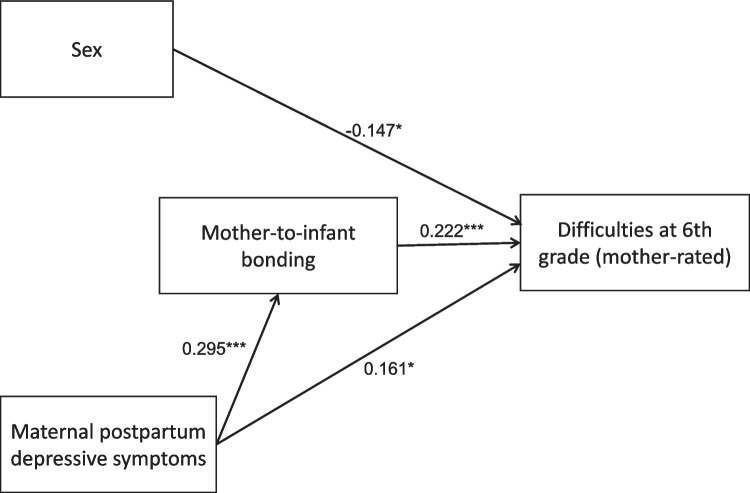
Path analysis for parent-rated SDQ scores after excluding non-mother respondents. Path analysis for parent-rated SDQ scores after excluding non-mother respondents (*n* = 226). The structural equation model demonstrated an acceptable fit to the data, with *χ*^2^ = 0.551 (df = 1, *p* = 0.458), CFI = 1.000, TLI = 1.050, RMSEA = 0.000, SRMR = 0.015, *R*.^2^ = 0.119 for SDQ scores and 8.7% for MIBS-J scores. Values shown in the figure are standardized estimates. *p < 0.05, **p < 0.01, ***p < 0.001

Figure 3a and b and Tables 4 and 5 show the results of the path analysis. Significant direct effects of sex were observed on both parent- and self-rated SDQ scores, with boys being associated with higher SDQ scores (*β* = − 0.135, *p* = 0.035 and *β* = − 0.172, *p* = 0.005, respectively). Maternal postpartum depressive symptoms had a significant effect on MIBS-J scores (*β* = 0.348, *p* < 0.001). MIBS-J scores had a significant effect on both parent- and self-rated SDQ scores (*β* = 0.219, *p* < 0.001 and *β* = 0.182, *p* = 0.005, respectively). Maternal postpartum depressive symptoms had a significant direct effect on parent-rated SDQ scores (*β* = 0.146, *p* = 0.035), but not on self-rated SDQ scores (*β* = 0.118, *p* = 0.107). Maternal postpartum depressive symptoms had a significant indirect effect on both parent- and self-rated SDQ through MIBS-J (*β* = 0.076, *p* = 0.020 and *β* = 0.064, *p* = 0.047, respectively). These models demonstrated an acceptable fit to the data, as described in figure legends. When the model was applied to parent-rated SDQ scores after excluding non-mother respondents, the significance level remained consistent across all tests (Fig. 4 and Table 6).

**Table 4:** Path analysis results using parent-rated SDQ scores

	Unstandardized estimate	S.E	95%CI lower	95%CI upper	Standardized estimate	*P* value
SDQ (parent-rated) ← Maternal depression	2.034	0.963	0.195	3.987	0.146	0.035
SDQ (parent-rated) ← Sex	− 1.422	0.670	− 2.737	− 0.097	− 0.135	0.034
SDQ (parent-rated) ← Mother-to-infant bonding	0.491	0.146	0.166	0.742	0.219	< 0.001
Mother-to-infant bonding ← Maternal depression	2.172	0.530	1.219	3.316	0.348	< 0.001
Indirect effect of maternal depression on SDQ (parent-rated) through mother-to-infant bonding	1.067	0.458	0.331	2.162	0.076	0.020
Total effect of maternal depression on SDQ (parent-rated)	3.101	0.984	1.244	5.159	0.222	0.002

*SDQ* Strengths and Difficulties Questionnaire, *EPDS* Edinburgh Postnatal Depression Scale, *SE* standard errors, *CI* confidence interval

**Table 5:** Path analysis results using self-rated SDQ scores

	Unstandardized estimate	S.E	95%CI lower	95%CI upper	Standardized estimate	*P* value
SDQ (self-rated) ← Maternal depression	1.799	1.115	− 0.313	4.135	0.118	0.107
SDQ (self-rated) ← Sex	− 1.983	0.708	− 3.416	− 0.623	− 0.172	0.005
SDQ (self-rated) ← Mother-to-infant bonding	0.446	0.158	0.165	0.774	0.182	0.005
Mother-to-infant bonding ← Maternal depression	2.172	0.530	1.216	3.326	0.348	< 0.001
Indirect effect of maternal depression on SDQ (self-rated) through mother-to-infant bonding	0.969	0.487	0.261	2.192	0.064	0.047
Total effect of maternal depression on SDQ (self-rated)	2.768	1.087	0.711	5.002	0.182	0.011

*SDQ* Strengths and Difficulties Questionnaire, *EPDS* Edinburgh Postnatal Depression Scale, *SE* standard errors, *CI* confidence interval

**Table 6:** Path analysis results using mother-rated SDQ scores

	Unstandardized estimate	S.E	95%CI lower	95%CI upper	Standardized estimate	*P* value
SDQ (mother-rated) ← Maternal depression	2.274	1.003	0.311	4.244	0.161	0.023
SDQ (mother-rated) ← Sex	− 1.541	0.685	− 2.860	− 0.204	− 0.147	0.024
SDQ (mother-rated) ← Mother-to-infant bonding	0.515	0.156	0.151	0.764	0.222	< 0.001
Mother-to-infant bonding ← Maternal depression	1.796	0.520	0.861	2.926	0.295	< 0.001
Indirect effect of maternal depression on SDQ (mother-rated) through mother-to-infant bonding	0.925	0.431	0.269	2.022	0.065	0.032
Total effect of maternal depression on SDQ (mother-rated)	3.199	1.034	1.128	5.233	0.226	0.002

*SDQ* Strengths and Difficulties Questionnaire, *EPDS* Edinburgh Postnatal Depression Scale, *SE* standard errors, *CI* confidence interval

**Fig. 5:**
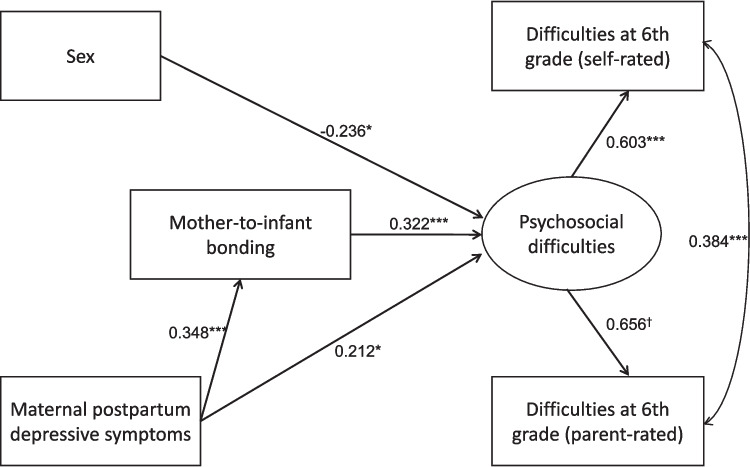
Unified path analysis modeling psychosocial difficulties in sixth-grade children. The structural equation model (*n* = 245) demonstrated a good fit to the data. The chi-square statistic was non-significant (*χ*2 = 2.546, df = 2, *p* = 0.280), indicating no significant deviation between the model and the observed data. The comparative fit index (CFI = 0.997) and Tucker-Lewis index (TLI = 0.987) exceeded the commonly accepted thresholds for good fit, while the root mean square error of approximation (RMSEA = 0.033) and standardized root mean square residual (SRMR = 0.023) suggested a close approximation of the model to the observed data. The model accounted for 43.0% of the variance in parent-rated SDQ scores, 36.4% in self-rated SDQ scores, 12.1% in mother–child bonding scores, and 26.1% in psychosocial difficulties, as indicated by the respective *R*^2^ values. Numbers indicate standardized estimate values. *P < 0.05, **P < 0.01, ***P < 0.001, ^†^not tested for statistical significance as it represents a factor loading

Additionally, an analysis using a latent variable (Fig. 5 and Table 7) showed that maternal postpartum depressive symptoms had a significant direct effect on child psychosocial difficulties (*β* = 0.212, *p* = 0.044). Furthermore, maternal postpartum depressive symptoms significantly predicted lower levels of mother-to-infant bonding (*β* = 0.348, *p* < 0.001), which in turn significantly predicted higher levels of child psychosocial difficulties (*β* = 0.322, *p* < 0.001). The indirect effect of maternal postpartum depressive symptoms on child psychosocial difficulties through mother-to-infant bonding was significant (*β* = 0.112, *p* = 0.021). The mediation effect size was 1.03, indicating that 34.6% of the total effect of maternal postpartum depressive symptoms on child psychosocial difficulties was mediated through mother-to-infant bonding. The model demonstrated a good fit to the data, as described in the figure legend. The model accounted for 43.0% of the variance in parent-rated SDQ scores, 36.4% in self-rated SDQ scores, 12.1% in mother-to-infant bonding scores, and 26.1% in psychosocial difficulties. The results of an additional analysis, including maternal age at delivery as a covariate, are presented in Supplementary Table 2 and Supplementary Fig. 1.

**Table 7:** Unified path analysis modeling psychosocial difficulties in sixth-grade children

	Unstandardized estimate	S.E	95%CI lower	95%CI upper	Standardized estimate	*P* value
Psychosocial difficulties ← SDQ (parent-rated)	1.000	0.000	1.000	1.000	0.656	NA
Psychosocial difficulties ← SDQ (self-rated)	1.004	0.278	0.600	1.635	0.603	0.000
Psychosocial difficulties ← Maternal depression	1.940	0.962	0.086	3.859	0.212	0.044
Psychosocial difficulties ← Sex	− 1.636	0.642	− 2.920	− 0.401	− 0.236	0.011
Psychosocial difficulties ← Mother-to-infant bonding	0.473	0.141	0.171	0.720	0.322	< 0.001
Mother-to-infant bonding ← Maternal depression	2.172	0.529	1.185	3.246	0.348	< 0.001
Indirect effect of maternal depression on Psychosocial difficulties through mother-to-infant bonding	1.027	0.445	0.326	2.108	0.112	0.021
Total effect of maternal depression on Psychosocial difficulties	2.968	1.001	0.995	4.980	0.324	0.003

*SDQ* Strengths and Difficulties Questionnaire, *EPDS* Edinburgh Postnatal Depression Scale, *SE* standard errors, *CI* confidence interval

## Discussion and conclusions

This study showed that postpartum mother-to-infant bonding was significantly associated with children’s difficulties in sixth grade. Maternal depressive symptoms were also linked to these difficulties, mediated by mother-to-infant bonding. The direct effect of maternal depression was significant for parent-rated SDQ scores but not for self-rated scores.

The prevalence of positive EPDS scores in this study aligned with the 15.1% prevalence of postpartum depression reported among Japanese women (Tokumitsu et al. 2020). Similarly, the mean (SD) MIBS-J score in this study closely resembled the reported range of 1.5–2.1 in previous studies of women at 1 month postpartum in Japan (Hashijiri et al. 2021; Yoshida et al. 2012). Mean SDQ scores of children also matched those in nationwide studies (Moriwaki and Kamio 2014). Consistent with previous studies in Japan (Matsuishi et al. 2008; Moriwaki and Kamio 2014), boys showed higher SDQ scores than girls, primarily due to conduct problems and hyperactivity/inattention subscales.

Maternal postpartum depressive symptoms were significantly associated with mother-to-infant bonding, consistent with findings by Yoshida et al. (2012), which highlight how depression-induced rejection and anger can disrupt parenting. Therefore, future research should investigate the specific depressive symptoms that affect bonding in larger sample populations.

Consistent with previous studies (Blair et al. 2023; Stein et al. 2014), the present study showed that postpartum depression was negatively associated with children’s future well-being. Path analysis revealed that maternal depression indirectly influenced both parent- and self-rated SDQ scores through bonding, underscoring bonding’s mediating role. Consistent with a previous study in Japanese adolescents (Kawabe et al. 2021), self-rated SDQ scores were significantly higher than parent-rated scores. However, parent-rated SDQ scores were more strongly associated with maternal depression, potentially reflecting biased perceptions among mothers with a history of postpartum depression, who are at a significantly higher risk of recurrent depressive episodes later in life (Bloch et al. 2024; Vliegen et al. 2014).

Previous studies showed that early bonding difficulties correlate with social-emotional problems in children aged 12–16 months (Behrendt et al. 2019) and at two years of age (Rusanen et al. 2024). Moreover, Englund et al (2011) showed that attachment security at 12 to 18 months correlated with friendship security at 16 years, as well as with global adaptive functioning at 28 years of age. Our findings expand this evidence, showing that early bonding issues affect children’s sixth-grade difficulties.

Future research should prioritize developing interventions that enhance postpartum mother-to-infant bonding, as this may help mitigate long-term psychosocial difficulties in children. The present study highlights the importance of addressing bonding difficulties as a potential treatment focus for mothers experiencing postpartum depression. Further exploration of the mechanisms underlying these associations is essential for designing targeted interventions to support affected families. Using a well-defined cohort and validated tools (EPDS, MIBS-J, SDQ), this study offers a robust model for examining maternal mental health and child outcomes. Structural equation modeling effectively clarified complex pathways, providing a methodological framework for future research.

Given the limited research on the biological correlates of mother-infant interactions in women with postpartum depression, this area warrants further research. Studies have consistently demonstrated that the intergenerational transmission of psychopathology affects not only offspring mental health but also their biology and predisposition to disease (Bind 2022). These findings underscore the need for an integrated approach to better understand and address these complex dynamics.

Several limitations of this study should be considered. First, the study relied on self-reported questionnaires and lacked clinical diagnoses. Second, the study was conducted in a single region of Japan, potentially limiting generalizability. Additionally, the follow-up rate was low, raising concerns about potential selection bias. However, the EPDS, MIBS-J, and SDQ scores were consistent with nationwide data (Hashijiri et al. 2021; Moriwaki and Kamio 2014; Tokumitsu et al. 2020; Yoshida et al. 2012), suggesting that, with respect to these scores, the study participants were representative of the general population in Japan. Third, unexamined confounders, such as socioeconomic status, family composition, and parental education, and maternal neuropsychiatric diagnoses may influence the findings. Additionally, adverse childhood experiences, including parental divorce, are known to be strongly associated with externalizing and internalizing behaviors in children (Hunt et al. 2017) and should be considered in future research. The impact of neuropsychiatric diagnoses in both mothers and children should also be investigated in future studies.

Fourth, this study lacks data related to genetic factors, which may partly explain the association between postpartum EPDS scores and child development outcomes. Shared genetic predispositions for mental health traits could contribute to both maternal depressive symptoms and child behavioral or emotional difficulties (Jami et al. 2021). The absence of genetic information and detailed maternal mental health history limits our ability to fully disentangle genetic and environmental influences. Future research incorporating genetic data and longitudinal tracking of maternal mental health is essential to address this limitation comprehensively.

Finally, this study lacked a formal sample size calculation prior to data collection. Instead, we aimed to include all eligible participants within the cohort during the defined timeframe. The statistically significant associations observed suggest that our sample size was sufficient to detect meaningful effects. While this approach maximized the sample size and allowed for a comprehensive examination of the relationships between EPDS, MIBS-J, and SDQ scores, it may have limited the ability to detect small effect sizes. Future studies should consider conducting formal power analyses to ensure optimal sample sizes for detecting smaller effect sizes with greater precision.

In conclusion, this study demonstrates that postpartum mother-to-infant bonding difficulties are significantly associated with children’s psychosocial difficulties in sixth grade. Maternal postpartum depressive symptoms were also found to influence children’s difficulties, with mother-to-infant bonding partially mediating this relationship. These findings not only underscore the enduring associations between early attachment experiences and children's developmental and emotional well-being but also extend theoretical frameworks by elucidating the mediating role of bonding in the context of maternal mental health.

## Supplementary Information

Below is the link to the electronic supplementary material.

Supplementary file1 (DOCX 48 KB)

Supplementary file2 (XLSX 12 KB)

Supplementary file3 (XLSX 12 KB)

Supplementary file4 (TIF 90 KB)

## Data Availability

For privacy and ethical reasons, data from this study can be made available by the corresponding author upon reasonable request, subject to the necessary approvals.
