# Supplementary material for: Postpartum maternal depression, mother-to-infant bonding, and their association with child difficulties in sixth grade
Source: Arch Womens Ment Health. 2025 Apr 15;28(5):1283–94. doi: 10.1007/s00737-025-01585-y (PMC12436542; doi:10.1007/s00737-025-01585-y)
Supplement: Supplementary file 1 — Supplementary file1 (DOCX 48 KB) [file 737_2025_1585_MOESM1_ESM.docx]

***Archives of Women's Mental Health***

**Postpartum maternal depression, mother-to-infant bonding, and their association with child difficulties in sixth grade**

Daimei Sasayama^1,2,3*^, Tomonori Owa^4^, Tetsuya Kudo^4^, Wakako Kaneko^4^, Mizuho Makita^2,3^, Rie Kuge^2,3^, Ken Shiraishi^1,2,3^, Tetsuo Nomiyama^5^, Shinsuke Washizuka^1^, Hideo Honda^2,3^

^1^ Department of Psychiatry, Shinshu University School of Medicine, Matsumoto, Nagano, 390-8621, Japan

^2^ Department of Child and Adolescent Developmental Psychiatry, Shinshu University School of Medicine, Matsumoto, Nagano, 390-8621, Japan

^3^ Mental Health Clinic for Children, Shinshu University Hospital, Matsumoto, Nagano, 390-8621, Japan

^4^ Shinano Medical Welfare Center, Shimo-Suwa, Nagano, 393-0093, Japan

^5^ Department of Preventive Medicine and Public Health, Shinshu University School of Medicine, Matsumoto, Nagano, 390-8621, Japan

*Corresponding author:

Daimei Sasayama

Email: [sasayama@shinshu-u.ac.jp](mailto:sasayama@shinshu-u.ac.jp)

**Supplementary Methods**

***Study Resources***

Okaya, a city in central Japan with a population of 45,872, as of January 1, 2024, was selected as the research setting for its commitment to improving maternal and child health services, particularly early identification of developmental delays and suboptimal caregiving during health assessments. Mothers giving birth in Okaya are routinely administered the Edinburgh Postnatal Depression Scale (EPDS) and Mother-to-Infant Bonding Scale-Japanese version (MIBS-J) at postnatal health checkups approximately 2 weeks to 1 month after delivery. In this study, EPDS and MIBS-J data from postnatal health checkups were analyzed for mothers of children born between April 2, 2009, and April 1, 2012.

In Japan, the fiscal year begins in April. Children whose mothers’ EPDS and MIBS-J scores were obtained were enrolled in sixth grade at age 11 during fiscal years 2021–2023. The parent- and self-rated versions of the Japanese version of the SDQ were distributed to all sixth graders and their caregivers in public elementary schools in Okaya in November of 2021, 2022, and 2023. An information sheet detailing the study was enclosed with the questionnaire. Only caregivers who consented for both themselves and their child to participate were asked to complete the questionnaires and allow their children to do the same. Consent was confirmed by the return of the questionnaires using the enclosed envelope. Participants were offered a 1000-yen gift card upon completion. The inclusion criteria were: (1) both child- and parent-completed SDQ data were available, and (2) the mother’s EPDS and MIBS-J data were accessible from the postpartum health checkup records. The exclusion criteria were: (1) participants with invalid SDQ data, and (2) children from multiple births. The study was approved by the ethics committee of the Shinshu University School of Medicine (approval number 5129).

***Clinical Scales***

*Edinburgh Postnatal Depression Scale (EPDS), Japanese version*

Maternal depressive symptoms were assessed using the EPDS, a 10-item self-rating scale designed to screen for postpartum depression in community samples. The EPDS data were collected during postnatal health checkups in Okaya, administered to all mothers approximately 2 weeks to 1 month postpartum. Each item is rated on a 4-point Likert scale, with response options ranging from minimal endorsement (e.g., "Not at all," "Never") to high endorsement (e.g., "Yes, very often," "Yes, most of the time"). The Japanese version of the EPDS has shown a sensitivity of 75% and specificity of 93% in a Japanese community sample, using a cut-off point of ≥ 9 (Okano et al., 1996). Its psychometric properties have been thoroughly examined (Kubota et al., 2014). Mothers with EPDS scores of 9 or above were classified as having postpartum depressive symptoms, whereas those with scores below 9 were regarded as not having relevant symptoms.

The ten items in EPDS are as follows (Cox et al., 1987):

1. I have been able to see the funny side of things.
2. I have looked forward with enjoyment to things.
3. I have blamed myself unnecessarily when things went wrong.
4. I have been worried for no good reason.
5. I have felt scared or panicky for no very good reason.
6. Things have been getting on top of me.
7. I have been so unhappy that I have had difficulty sleeping (not because of the baby).
8. I have felt sad and miserable.
9. I have been so unhappy that I have been crying.
10. The thought of harming myself has occurred to me.

*Mother-to-Infant Bonding Scale-Japanese version (MIBS-J)*

The MIBS-J, administered during postnatal health checkups in Okaya approximately 2 weeks to 1 month postpartum, was used to evaluate mother-child bonding. The MIBS-J is based on the modified Kumar’s Mother-to-Infant Bonding Questionnaire (MIBQ) (Kumar, 1997), which originally consisted of nine items derived from mothers’ narrative accounts and was later revised to a ten-item scale (Yoshida et al., 2012). The MIBS-J is a ten-item questionnaire scored on a 4-point Likert scale. The total score ranges from 0 and 30, with higher scores indicating poorer bonding (Yoshida et al., 2012).

Cutoff scores for the MIBS-J have not been established. However, a validation study demonstrated reasonable internal consistency (Cronbach’s alpha = 0.71 for items in the lack of affection subscale and 0.51 for items in the anger and rejection subscale), a significant positive correlation with EPDS scores (Pearson’s *r* = 0.514 at one month postpartum), and high test-retest reliability (Pearson’s *r* = 0.439–0.530 for scores at one and four months postpartum) (Yoshida et al., 2012).

The ten items in MIBS-J are as follows (Yoshida et al., 2012):

1. Feel loving towards my baby
2. Feel scared or panicky when I have to do something for my baby
3. Feel resentful towards my baby
4. Feel nothing towards my baby
5. Feel angry with my baby
6. Enjoy doing things with my baby
7. Wish my baby was different
8. Feel protective towards my baby
9. Wish I did not have my baby
10. Feel close to my baby

Stre*ngths and Difficulties Questionnaire*

The Strengths and Difficulties Questionnaire (Goodman, 2001) is a widely used tool for assessing emotional and behavioral difficulties in children and adolescents. In this study, the Japanese versions of both the parent-rated and youth-self-rated SDQ were utilized. The SDQ consists of 25 items divided into five subscales: emotional symptoms, conduct problems, hyperactivity, peer problems, and prosocial behaviors. Each subscale comprises five items rated on a 3-point Likert scale: "Not true" (0), "Somewhat true" (1), and "Certainly true" (2). The total difficulties score is derived from the sum of the first four subscales, ranging from 0 to 40, with higher scores indicating greater difficulties.

The Japanese version of the SDQ has been shown to have a high internal consistency (Cronbach’s alpha = 0.81 for parent-rated score), moderate inter-rater reliability (Spearman’s *ρ* = 0.40 when parent-rated and teacher-rated were compared), high test-retest reliability (Spearman’s *ρ* = 0.79 for parent-rated score), and moderate to strong correlations with corresponding subscales of the Child Behavioral Checklist and the ADHD-rating scale-IV (Moriwaki and Kamio, 2014).

The 25 items in parent-rated SDQ are as follows:

1. Considerate of other people's feelings
2. Restless, overactive, cannot stay still for long
3. Often complains of headaches, stomach-aches or sickness
4. Shares readily with other youth, for example CD’s, games, food
5. Often loses temper
6. Would rather be alone than with other youth
7. Generally well behaved, usually does what adults request
8. Many worries or often seems worried
9. Helpful if someone is hurt, upset or feeling ill
10. Constantly fidgeting or squirming
11. Has at least one good friend
12. Often fights with other youth or bullies them
13. Often unhappy, depressed or tearful
14. Generally liked by other youth
15. Easily distracted, concentration wanders
16. Nervous in new situations, easily loses confidence
17. Kind to younger children
18. Often lies or cheats
19. Picked on or bullied by other youth
20. Often offers to help others (parents, teachers, children)
21. Thinks things out before acting
22. Steals from home, school or elsewhere
23. Gets along better with adults than with other youth
24. Many fears, easily scared
25. Good attention span, sees chores or homework through to the end

The 25 items in self-rated SDQ are as follows:

1. I try to be nice to other people. I care about their feelings
2. I am restless, I cannot stay still for long
3. I get a lot of headaches, stomach-aches or sickness
4. I usually share with others, for example CD’s, games, food
5. I get very angry and often lose my temper
6. I would rather be alone than with people of my age
7. I usually do as I am told
8. I worry a lot
9. I am helpful if someone is hurt, upset or feeling ill
10. I am constantly fidgeting or squirming
11. I have one good friend or more
12. I fight a lot. I can make other people do what I want
13. I am often unhappy, depressed or tearful
14. Other people my age generally like me
15. I am easily distracted, I find it difficult to concentrate
16. I am nervous in new situations. I easily lose confidence
17. I am kind to younger children
18. I am often accused of lying or cheating
19. Other children or young people pick on me or bully me
20. I often offer to help others (parents, teachers, children)
21. I think before I do things
22. I take things that are not mine from home, school or elsew
23. I get along better with adults than with people my own age
24. I have many fears, I am easily scared
25. I finish the work I'm doing. My attention is good

*Statistical analysis*

MIBS-J and SDQ scores were treated as continuous variables. Because these scores are known to be non-normally distributed (Moriwaki and Kamio 2014; Yoshida et al. 2012), differences in continuous variables between two groups were assessed using the Mann–Whitney U test. Spearman’s correlation coefficient was used to assess correlations between variables, as it does not assume normality. A Wilcoxon signed-rank test was used to compare self-rated and parent-rated SDQ scores. These analyses were performed using Statistical Package for the Social Sciences version 26 (IBM Corp., Armonk, NY).

Path analysis was conducted to examine the relationships among sex, maternal postpartum depressive symptoms, mother-to-infant bonding, and difficulties in the sixth grade. The presence of maternal postpartum depressive symptoms and sex was entered as the predictor, MIBS-J scores as the mediator, and SDQ scores (self-rated or parent-rated) as the dependent variable. As a sensitivity analysis, the same model was applied to parent-rated SDQ scores, excluding non-mother respondents. Additionally, we modeled self-rated and parent-rated SDQ scores as indicators of a single latent variable “psychosocial difficulties” to capture their shared variance, enabling a unified representation of child difficulties in the analysis. An additional analysis was performed with maternal age included as a covariate. The structural equational modeling was specified using the package ‘lavaan’ version 0.6-14 (Rosseel 2012) in R version 4.4.0. To address the non-normality of SDQ and MIBS-J scores, the structural equation model was estimated using the maximum likelihood estimation method, and 5,000 bootstrap samples were used to generate bias-corrected 95% confidence intervals to account for potential sampling variability. The ‘sem’ function was used to fit the model to the data. The ‘fitMeasures’ function was used to evaluate model fit indices by using the chi-square statistic, comparative fit index (CFI), Tucker-Lewis index (TLI), root mean square error of approximation (RMSEA), and standardized root mean square residual (SRMR). All tests were two-tailed, and statistical significance was set at p < 0.05.

**References**

Cox JL, Holden JM, Sagovsky R (1987) Detection of postnatal depression. Development of the 10-item Edinburgh Postnatal Depression Scale. Br J Psychiatry 150:782-786. <https://doi.org/10.1192/bjp.150.6.782>

Goodman R (2001) Psychometric properties of the strengths and difficulties questionnaire. J Am Acad Child Adolesc Psychiatry 40:1337-1345. <https://doi.org/10.1097/00004583-200111000-00015>

Kubota C, Okada T, Aleksic B, Nakamura Y, Kunimoto S, Morikawa M, Shiino T, Tamaji A, Ohoka H, Banno N, Morita T, Murase S, Goto S, Kanai A, Masuda T, Ando M, Ozaki N (2014) Factor structure of the Japanese version of the Edinburgh Postnatal Depression Scale in the postpartum period. PLoS One 9:e103941. <https://doi.org/10.1371/journal.pone.0103941>

Kumar RC (1997) "Anybody's child": severe disorders of mother-to-infant bonding. Br J Psychiatry 171:175-181. <https://doi.org/10.1192/bjp.171.2.175>

Moriwaki A, Kamio Y (2014) Normative data and psychometric properties of the strengths and difficulties questionnaire among Japanese school-aged children. Child Adolesc Psychiatry Ment Health 8:1. <https://doi.org/10.1186/1753-2000-8-1>

Okano T, Murata M, Masuji F, Tamaki R, Nomura J, Miyaoka H, Kitamura T (1996) Validation and reliability of a Japanese version of the EPDS. Archives of Psychiatric Diagnostics and Clinical Evaluation 7:525-533

Yoshida K, Yamashita H, Conroy S, Marks M, Kumar C (2012) A Japanese version of Mother-to-Infant Bonding Scale: factor structure, longitudinal changes and links with maternal mood during the early postnatal period in Japanese mothers. Arch Womens Ment Health 15:343-352. <https://doi.org/10.1007/s00737-012-0291-1>
