# Supplementary figures and images for: Postpartum maternal depression, mother-to-infant bonding, and their association with child difficulties in sixth grade
Source: Arch Womens Ment Health. 2025 Apr 15;28(5):1283–94. doi: 10.1007/s00737-025-01585-y (PMC12436542; doi:10.1007/s00737-025-01585-y)

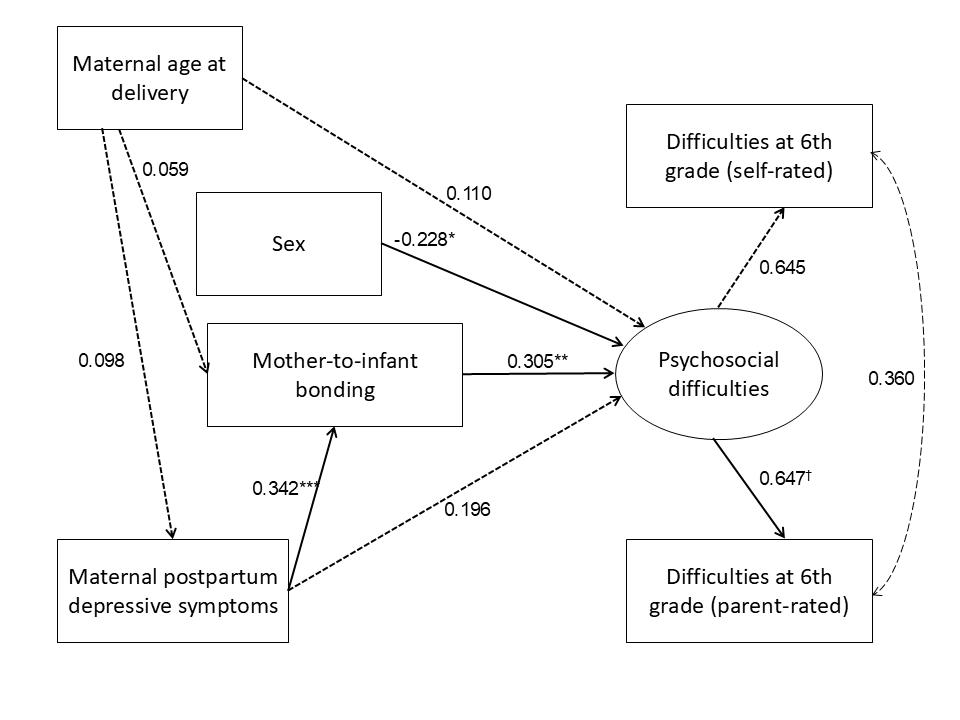

Supplement: Supplementary file 4 — Supplementary file4 (TIF 90 KB) [file 737_2025_1585_MOESM4_ESM.tif]
